# CD36 relative mean fluorescence intensity of CD105^+^ nucleated erythroid cells can be used to differentiate myelodysplastic syndrome from megaloblastic anemia

**DOI:** 10.1038/s41598-023-35994-9

**Published:** 2023-06-01

**Authors:** Yan Lu, Xuya Chen, Longyi Zhang

**Affiliations:** Clinical Laboratory, Dongyang People’s Hospital, 60 West Wuning Road, Dongyang, 322100 Zhejiang China

**Keywords:** Diagnostic markers, Myelodysplastic syndrome

## Abstract

This study aims to evaluate the differences in CD105^+^ nucleated erythroid cell (NEC) immunophenotypes between myelodysplastic syndrome (MDS) and megaloblastic anemia (MA) using multiparameter flow cytometry and to screen potential markers. We analyzed bone marrow sample data from 37 patients with MDS, 35 with MA, 53 with iron-deficiency anemia (anemic controls), and 35 without anemia (normal controls). Compared with normal controls, the MDS and MA groups showed a decrease in the proportion of CD117^+^CD105^+^NEC and the relative mean fluorescence intensity (RMFI) of CD71 in CD105^+^NEC, accompanied by an increase in the coefficient of variation (CV) of CD71 and CD36. Additionally, CD36 RMFI of CD105^+^NEC increased in the MA group. Compared with anemia controls, the MDS and MA groups showed a significant increase in CD36 CV of CD105^+^NEC, and the CD36 RMFI in the MA group increased while that in the MDS group decreased. The proportions of CD117^+^CD105^+^NEC, CD36 CV, and CD36 RMFI in CD105^+^NEC differed significantly between MDS and MA groups. Among them, CD36 RMFI had good diagnostic performance (area under the curve: 0.844, 95% confidence interval: 0.753–0.935). CD36 RMFI of CD105^+^NEC may be a helpful marker in differentiating MDS and MA using multiparameter flow cytometry.

## Introduction

Myelodysplastic syndrome (MDS) is an acquired heterogeneous clonal disease of hematopoietic stem cells that is characterized by dysregulated hematopoietic differentiation, cytopenia, and morphological dysplasia^1^. Megaloblastic anemia (MA) is a type of macrocytic anemia caused by altered deoxyribonucleic acid synthesis due to deficient vitamin B12 and/or folic acid. Typically, the nucleus and cytoplasm of the nucleated erythroid cells and granulocytes develop out of synchrony and undergo megaloblastic metamorphosis^2^. MDS and MA have similarities in certain clinical manifestations and laboratory tests. For instance, both MDS and MA can exhibit pancytopenia as well as dysplasia^3,4^. Therefore, the differentiation of MDS with MA is met with difficulty in certain situations.

In clinical practice, there are many methods (including bone marrow morphology, cytogenetic testing^5^, next-generation sequencing^6^, and folate or vitamin B12 levels) for the differential diagnosis of anemia due to MDS and MA. Although the accuracy of a single test method is limited, laboratories may not necessarily use multiple tests to distinguish MDS and MA due to the limitations of test conditions and costs. Immunophenotypic analysis by flow cytometry is well known to evaluate the differentiation and maturity of myeloid cells. However, it is not frequently used to differentiate between MDS and MA due to the absence of suitable markers.

Since anemia symptoms are present in most patients with MDS and MA, flow cytometry for the bone marrow immunophenotypic characterization of nucleated erythroid cells (NEC) may be a useful auxiliary technique. Existing studies have described the abnormal NEC immunophenotypes in patients with MDS, including increased coefficient of variation (CV) of CD36 and CD71 fluorescence intensities and decreased mean fluorescence intensity (MFI) of CD36 and CD71^7,8^. For patients with MA, the characterization of the NEC immunophenotype during erythropoiesis, differentiation, and maturation may be crucial; however, the expression pattern of the NEC immunophenotype has not yet been determined.

Currently, it is still controversial whether erythrolysis is required in the process of NEC detection by flow cytometry^9,10^. The strategy of avoiding erythrocyte lysis to prevent the decrease of NEC may not be suitable in practice for all laboratories. This is primarily due to the increased risk of blockage of instrument tubing. Therefore, the use of ammonium chloride (NH_4_Cl) in lysing mature red blood cells is routine for practical laboratory testing. However, it may affect the scattering parameters and ratio of the remaining cells^11^. The principle of NH_4_Cl lysis of erythrocytes is based on the presence of a large amount of carbonic anhydrase in erythrocytes^12^, whereas CD105^+^NEC does not have carbonic anhydrase. Therefore, the CD105^+^NEC population is not affected by NH_4_Cl lysis^13^.

To increase the comparability of data, the ratio between CD105^+^NEC MFI and lymphocyte MFI was used as relative mean fluorescence intensity (RMFI) in this study to replace MFI. This study aimed to evaluate the differences in CD105^+^ NEC immunophenotypes between MDS and MA using multiparameter flow cytometry and to screen potential flow cytometry markers to distinguish between the the two conditions.

## Results

### CD105^+^NEC immunophenotype in MDS

In total, 37 patients with MDS were included in this study. According to the World Health Organization criteria, the following different MDS subtypes were present: thirteen cases of MDS with multilineage dysplasia (MDS-MLD); three cases of MDS with single lineage dysplasia (MDS-SLD); three cases of MDS with ring sideroblasts and single lineage dysplasia (MDS-RS-SLD); twelve cases of MDS with excess blasts, 5–9% in the bone marrow, or 2–4% in the blood (MDS-EB-I); and six cases of MDS with excess blasts, 10–19% in the bone marrow, or 5–19% in the blood (MDS-EB-II).

**Figure 1:**
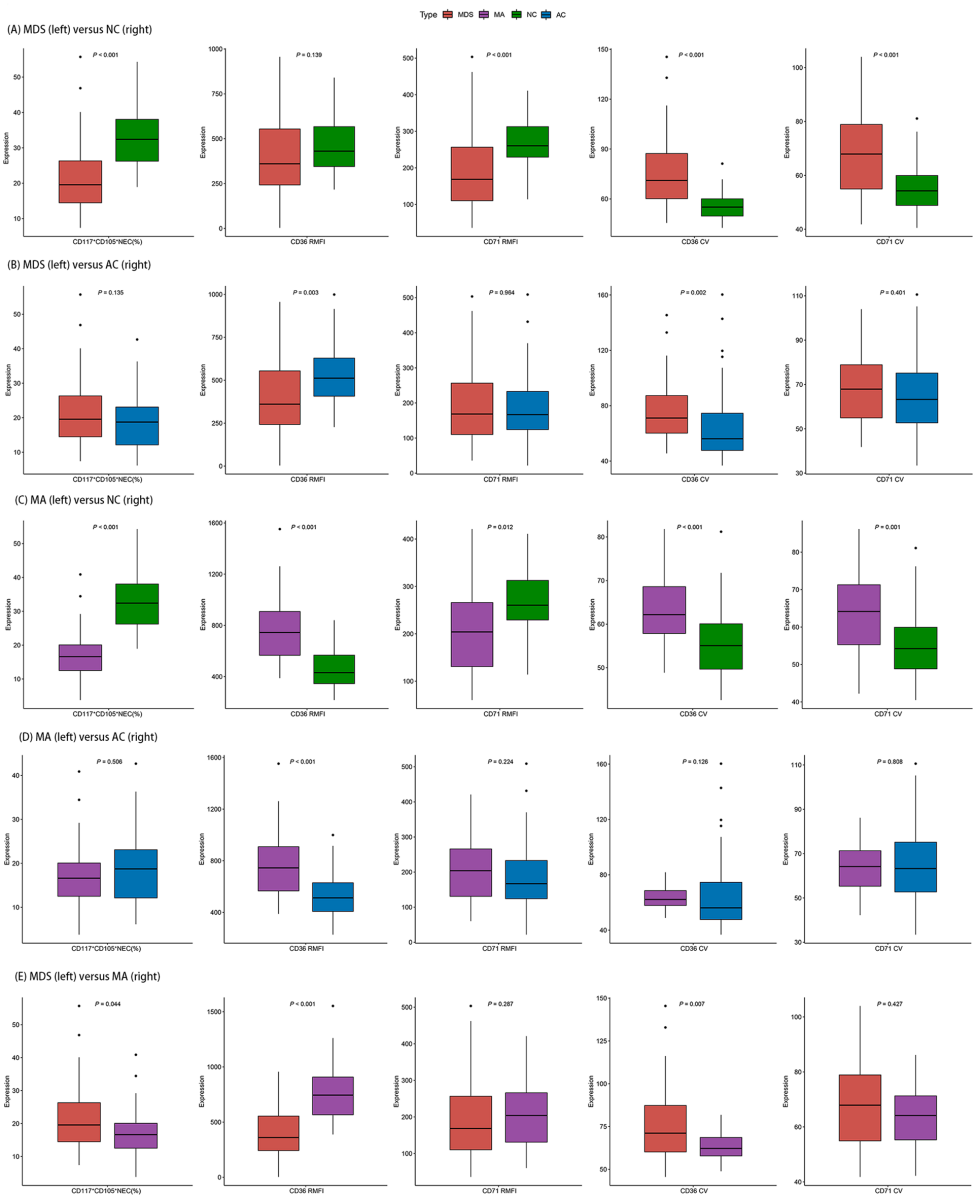
Differences in the immunophenotype of CD105^+^ nucleated erythroid cells in patients with MDS, MA, NC, and AC. (**A**) MDS versus NC; (**B**) MDS versus AC; (**C**) MA versus NC; (**D**) MA versus AC; (**E**) MDS versus MA. MDS: myelodysplastic syndrome; MA: megaloblastic anemia; NC: normal controls; AC: anemic controls; NEC: nucleated erythroid cells; RMFI: relative mean fluorescence intensity; CV: coefficient of variation.

As shown in Table 1 and Fig. 1A, the proportion of CD117^+^CD105^+^NEC (% of CD105^+^ NEC) decreased in MDS compared with normal controls (NC) (median 19.6 vs. 32.4, *P* < 0.001). In addition, CD71 RMFI of CD105^+^ NEC was significantly decreased in MDS (median 169 vs. 260, *P* < 0.001), accompanied by a marked increase in the CV of CD71 (median 67.9 vs. 54.2, *P* < 0.001) and CD36 (median 71.1 vs. 55.1, *P* < 0.001).

**Table 1:** CD105^+^NEC immunophenotyping in MDS, MA, NC, and AC.

	MDS (N = 37)	MA (N = 35)	NC (N = 35)	AC (N = 53)
CD117^+^CD105^+^ NEC (%)	19.6 [14.5–26.3]	16.6 [12.5–20.1]	32.4 [26.2–38.1]	18.7 [12.1–23.1]
CD36 RMFI	360 [242–554]	744 [566–908]	431 [345–567]	512 [407–629]
CD71 RMFI	169 [110–256]	204 [131–266]	260 [229–313]	167 [124–233]
CD36 CV	71.1 [60.2–87.3]	62.2 [57.9–68.6]	55.1 [49.7–60.1]	56.1 [47.7–74.6]
CD71 CV	67.9 [54.9–78.9]	64.2 [55.3–71.3]	54.2 [48.8–59.9]	63.3 [52.7–75.2]

*RMFI* relative mean fluorescence intensity, *MDS* myelodysplastic syndrome, *MA* megaloblastic anemia, *NC* normal controls, *AC* anemia controls, *CV* coefficient of variation, *NEC* nucleated erythroid cells.

Compared with anemia controls (AC), the CD36 CV in MDS significantly increased (median 71.1 vs. 56.1, *P* = 0.002) and CD36 RMFI of CD105^+^ NEC was significantly decreased (median 360 vs. 512, *P* = 0.003). However, there was no significant difference in CD117^+^CD105^+^NEC (% of CD105^+^NEC), CD71 CV, and CD71 RMFI between MDS and AC (Table 1, Fig. 1B).

### CD105^+^NEC immunophenotyping in MA

As shown in Table 1 and Fig. 1C, the proportion of CD117^+^CD105^+^NEC decreased in MA compared with NC (median 16.6 vs. 32.4, *P* < 0.001). In addition, both the CD36 RMFI and CD36 CV of CD105^+^NEC were significantly increased in MA (median 744 vs. 431, *P* < 0.001; median 62.2 vs. 56.1, *P* < 0.001). Furthermore, the CD71 RMFI of MA was decreased (median 204 vs. 260, *P* = 0.012), but the CD71 CV was significantly increased (median 64.2 vs. 54.2, *P* = 0.001) relative to NC.

Moreover, the CD36 RMFI of CD105^+^NEC in MA was significantly increased than AC (median 744 vs. 512, *P* < 0.001) (Table 1, Fig. 1D).

### Differential diagnosis between MDS and MA

As shown in Table 1 and Fig. 1E, CD36 RMFI of CD105^+^NEC showed significant differences in expression levels between MDS and MA (median 360 vs. 744, *P* < 0.001). Moreover, compared with MA, the proportion of CD117^+^CD105^+^NEC and CD36 CV of CD105^+^NEC was significantly increased in MDS. Supplementary Fig. 1 shows typical NEC immunophenotypes in MDS, MA, NC, and AC.

**Figure 2:**
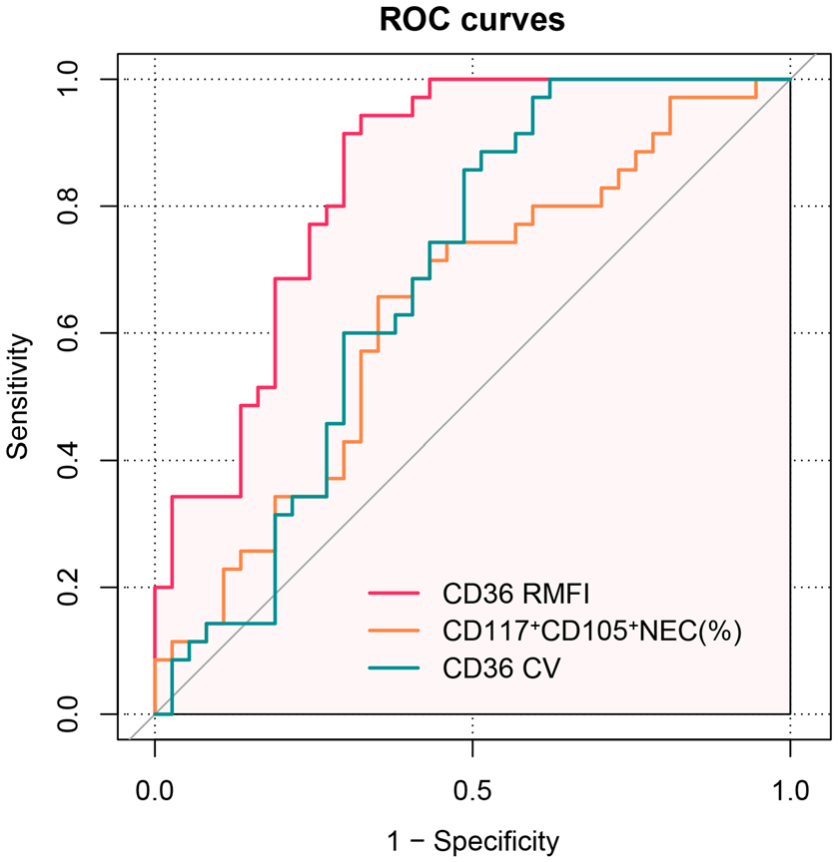
The receiver operating characteristic curves of CD117^+^CD105^+^ nucleated erythroid cells (% of CD105^+^nucleated erythroid cells), CD36 CV, and CD36 RMFI.

To differentiate between MDS and MA, the diagnostic performance of CD117^+^CD105^+^NEC (% of CD105^+^NEC), CD36 CV, and CD36 RMFI is shown in Fig. 2. In Table 2, we found that CD36 RMFI has good diagnostic performance (area under the curve (AUC): 0.844, 95% CI: 0.753–0.935), the best cut-off value for which was 474.36. In addition, the sensitivity, specificity, accuracy, positive predictive value (PPV), and negative predictive value (NPV) of CD36 RMFI to differentiate MDS and MA were 94.3%, 67.6%, 80.6%, 73.3% and 92.6%, respectively. CD117^+^CD105^+^NEC (% of CD105^+^NEC) and CD36 CV also had certain differential diagnostic values, but their diagnostic performances were inferior to that of CD36 RMFI (AUC: 0.638 vs. AUC: 0.685 vs. AUC: 0.844).

**Table 2:** Diagnostic performance of CD117^+^CD105^+^NEC (% of CD105^+^NEC), CD36 CV, and CD36 RMFI to differentiate between MDS and MA.

	CD36 RMFI	CD117^+^CD105^+^NEC (%)	CD36 CV
Area under the curve	0.844	0.638	0.685
95% CI lower	0.753	0.509	0.559
95% CI upper	0.935	0.767	0.810
Cut-off value	474.36	17.59	83.11
Sensitivity	94.3%	64.9%	37.8%
Specificity	67.6%	65.7%	100.0%
Accuracy	80.6%	65.3%	72.2%
Positive predictive value	73.3%	66.7%	70.7%
Negative predictive value	92.6%	63.9%	74.2%

*CI* confidence interval, *CV* coefficient of variation, *RMFI* relative mean fluorescence intensity, *MDS* myelodysplastic syndrome, *MA* megaloblastic anemia, *NEC* nucleated erythroid cells.

**Figure 3:**
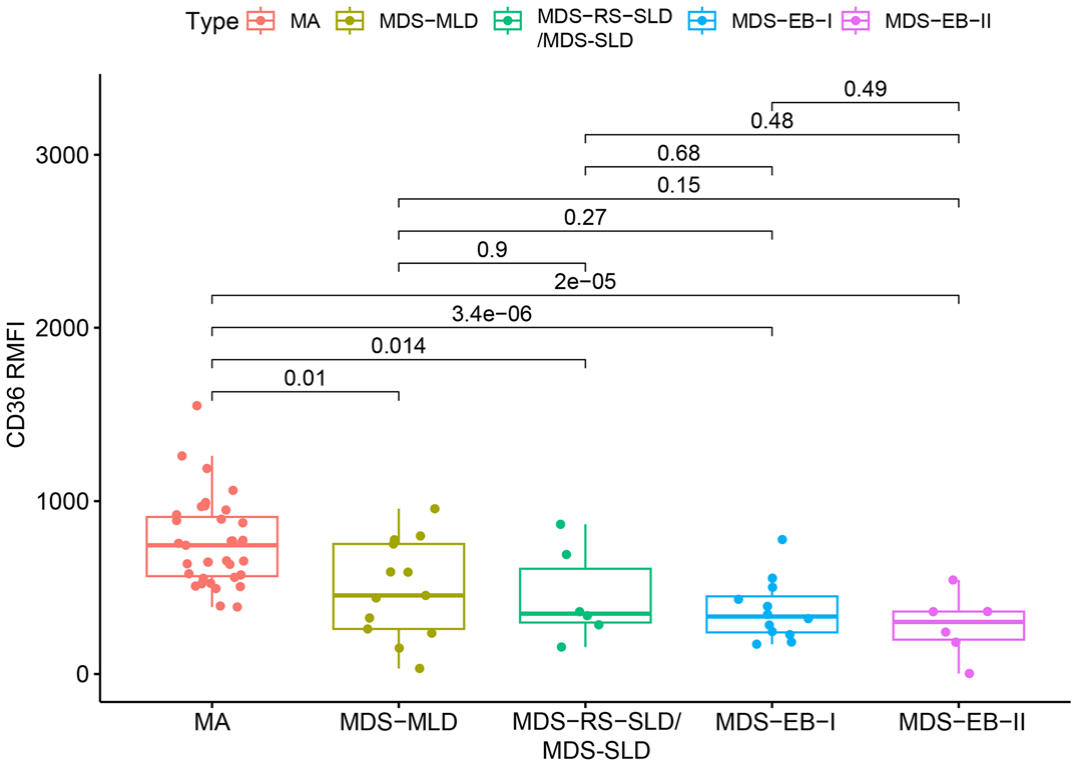
Comparison between MA and different subtypes of MDS. MA: megaloblastic anemia; MDS-MLD: MDS with multilineage dysplasia; MDS-SLD: MDS with single lineage dysplasia; MDS-RS-SLD: MDS with ring sideroblasts and single lineage dysplasia; MDS-EB-I: MDS with excess blasts, 5–9% in the bone marrow, or 2–4% in the blood; MDS-EB-II: MDS with excess blasts, 10–19% in the bone marrow, or 5–19% in the blood.

Moreover, as shown in Fig. 3, the CD36 RMFI in CD105^+^NEC of MA was significantly different from each subtype of MDS, while the difference between each subtype of MDS was not significant.

## Discussion

In this study, we used multiparameter flow cytometry to evaluate the erythroid development of MDS and MA. Interestingly, the present study found for the first time that CD36 RMFI of CD105^+^NEC was differentially expressed between MDS and MA, which may be a robust immunophenotypic marker for differential diagnosis.

The expression of the CD36 antigen starts from early erythroid progenitor cells and disappears at the reticulocyte stage^14^. CD71, also known as transferrin receptor I, also appears at elevated and then decreasing expression levels from early erythroid progenitors and is absent in mature erythrocytes^15^. As immunophenotypic markers, most studies focused on the role of CD36 and CD71 in the differential diagnosis of MDS and non-clonal cytopenia. Studies have shown that MFI and CV of CD36 and CD71 can be used as differential indicators between patients with MDS and non-clonal cytopenias^10,16,17^. Although RMFI rather than MFI was used in this study, similar results were obtained. This study also concluded that the CD105^+^NEC of patients with MDS showed decreased CD71 RMFI and increased CV of CD36 and CD71, compared with the NC group. In addition, in this study, the CD36 RMFI of CD105^+^NEC was not statistically different between the MDS and NC groups. However, compared with the AC group, in the MDS group, the CD36 RMFI and CV of CD105^+^NEC were significantly different, while the CD71 RMFI and CV were not. Therefore, we realize the importance of the control group setting, which could explain the difference in results. Furthermore, this study demonstrated that although both IDA and MA exhibited non-clonal erythrocytopenia, the immunophenotype of NEC was heterogeneous. Consequently, other studies combined several different types of anemia into a group, which may have contributed to the discrepancy in the results.

The combined analyses of CD105 and CD117 can more accurately categorize erythroid progenitor cells^18^. Although previous studies have shown that the percentage of CD117^+^ erythroid abnormalities (obtained by flow cytometry) can be used for the diagnosis of MDS, the reduced proportion of CD117^+^ expression in CD105^+^NEC in patients with MA is the first to be proposed^7,8^. CD105^+^CD117^+^ cells, which represent the most immature erythroid cells, were reduced in proportion for both patients with MDS and MA, indicating that an abnormal erythropoiesis pattern is present in both. Therefore, the diagnostic performance of CD105^+^CD117^+^ cells as a marker for differentiating MDS and MA is not outstanding.

In the present study, we found that MDS and MA exhibit different NEC immunophenotypes, which can be more visualized by CD105/CD71, CD105/CD36, and CD105/CD117 erythroid differentiation trajectory maps. Supplementary Fig. 1 showed the differences in erythroid maturation patterns between MDS and MA. Using the expression of CD36 and CD71 in the NC group as a reference, the expression of CD71 is somewhat similar in MDS and MA, while the expression of CD36 is significantly different. In addition, in the case of unifying the number of CD105^+^NEC in this study, it is not difficult to see that the proportion of late erythroblasts (CD117^−^CD105^−^NEC) in MA is significantly lower, which may be caused by the nuclear development disorder in MA.

This study has several advantages. First, studies based on the immunophenotype of CD105^+^ NEC can reduce the impact of NH4Cl lysis on NEC analysis and facilitate routine laboratory operations. Second, the study confirmed a differential immunophenotype of CD105^+^ NEC in patients with MDS and characterized this cell population in the context of MA for the first time. Third, this study replaced MFI with RMFI, which increased the robustness and repeatability of the analysis, and to some extent can solve the problem of result deviation caused by different laser power and detector sensitivity. Meanwhile, there are limitations in the present study. The current results are mainly based on single-center small sample studies. Due to an insufficient sample size, we have not further explored the specific immunophenotypes of different subtypes of MDS. In addition, the identification between MA and MDS without classical dysplastic changes may be more challenging. Moreover, although CD36 RMFI of CD105^+^NEC may help distinguish between MA and MDS, further exploration is needed to differentiate MDS from other non-clonal anemia. And a multicenter study with a larger sample size and subgroup analysis is needed for further analysis.

This study characterized the immunophenotype of CD105^+^NEC in MDS and MA using multiparameter flow cytometry. Furthermore, CD36 RMFI of CD105^+^NEC was a potentially helpful marker for differentiating MDS and MA.

## Methods

### Patients

Since 2020, flow cytometry evaluation of the immunophenotype of bone marrow nucleated erythroid cells has been included as routine workup in the diagnosis of patients with hematological diseases in our hospital. For this study, bone marrow aspirate was collected before the patient was diagnosed and treated. We analyzed bone marrow sample data from 37 patients with MDS and 35 patients with MA who were admitted to Dongyang People's Hospital between November 2020 and January 2023. Additionally, 53 patients with iron deficiency anemia (IDA) and 35 patients without anemia were included as AC and NC, respectively. The diagnostic criteria for the patients were as follows: (1) MDS: diagnostic criteria per those of the World Health Organization (2016)^19^; (2) MA: deficient vitamin B12 or folic acid levels and bone marrow cell morphology showing obvious megaloblastic metamorphosis; (3) IDA: diagnosis made by two hematology clinicians considering cell morphology, serum iron level, and bone marrow cytology iron staining; (4) NC: patients with normal red blood cell count, morphology, and hemoglobin, including patients with leukopenia or leukocytosis with a suspected hematologic disease but unproven after a thorough medical examination (n = 12), patients with confirmed extramedullary lymphoma without bone marrow involvement (n = 4), and patients with platelet-limited disorders (thrombocytosis (n = 12) or thrombocytopenia (n = 7)).

This study was noninterventional. Data for all patients were obtained through retrospective retrieval and were anonymized for analysis. The study was approved by the institutional review board (IRB) of Dongyang People's Hospital. Since this was a retrospective study, the IRB of Dongyang People's Hospital waived the requirement for informed consent of the study subjects. All methods of this study were performed in accordance with the relevant guidelines and regulations.

### Flow cytometry analysis

**Figure 4:**
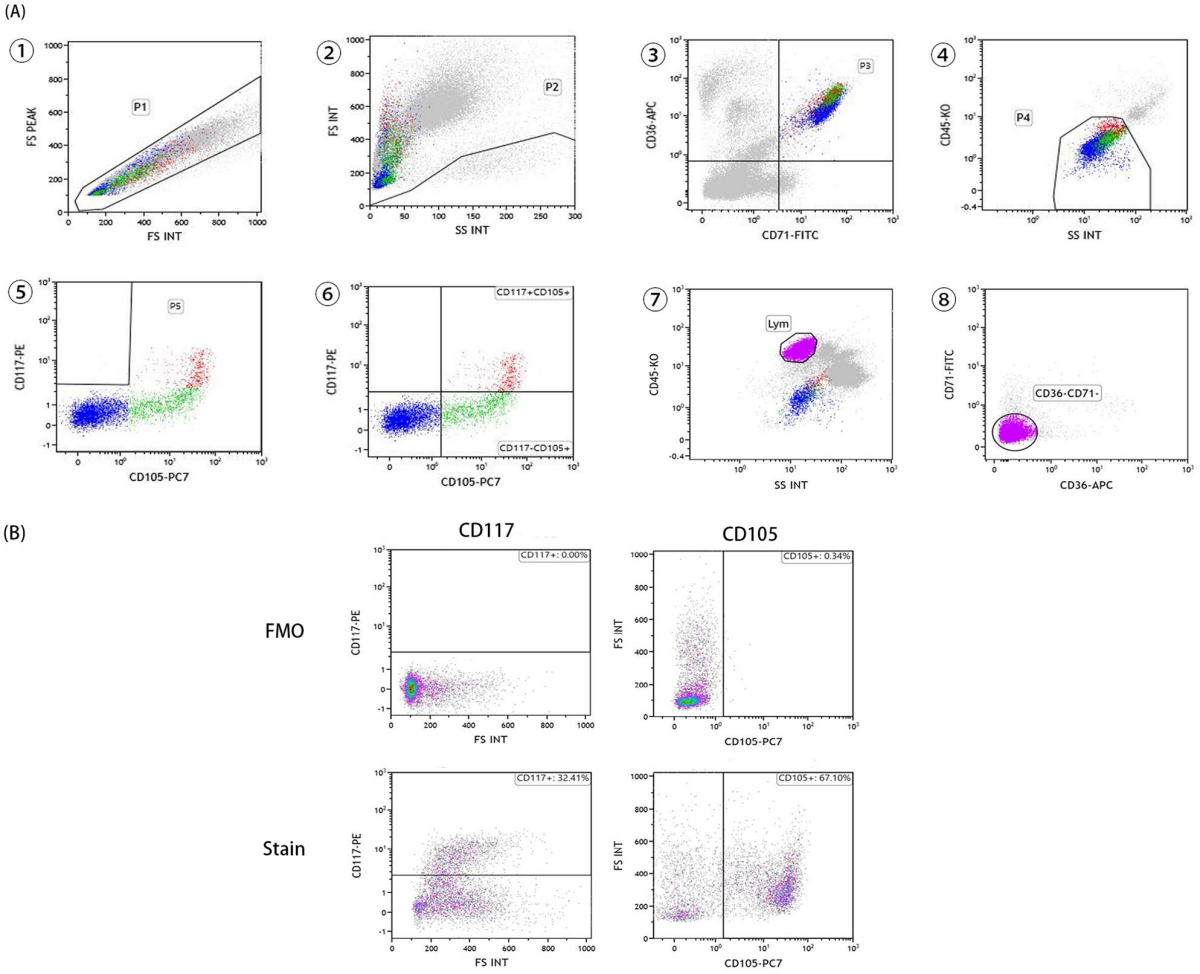
Gating strategy for measuring CD105^+^ nucleated erythroid cell immunophenotype. (A) After removing adhesions and fragments (①–②), all nucleated erythroid cells (③–④) were selected according to CD36/CD71 and CD45/SS, then nucleated erythroid cells were grouped according to the expression of CD117 and CD105 (⑤–⑥). Finally, according to CD45/SS and CD36/CD71, lymphocytes were selected for the calculation of relative average fluorescence intensity (⑦–⑧). (B) FMO controls for CD117 and CD105 were evaluated. FMO: fluorescence minus one.

Bone marrow samples anticoagulated with heparin were processed using a standardized surface fluorescent antibody staining technique within 24 h after bone marrow aspiration. Briefly, samples were washed twice with phosphate-buffered saline (PBS) and resuspended, and 100 µl of the sample was taken to bind premixed antibodies. Then, 0.5 ml OptiLyse C lysate (Beckman Coulter, USA) was added and left for 15 min to allow adequate lysis of erythrocytes. Finally, it was washed with PBS and resuspended. Surface dye combinations for NEC immunophenotyping were as follows: CD71-FITC (clone YDJ1.2.2; Beckman Coulter, CA, USA), CD117-PE (clone 104D2D1; Beckman Coulter), CD105-PE-Cy7 (clone TEA3/17.1.1; Beckman Coulter), CD36-APC (clone FA6.152; Biolegend, CA, USA), and CD45-KrO (clone J.33; Beckman Coulter). A ten-color flow cytometer (Naivos; Beckman Coulter) was used for the collection and detection of NEC. Test results were analyzed using the accompanying colleague Kaluza software (version 2.0, Beckman Coulter). The specific gating strategy is shown in Fig. 4A. The expression threshold of CD105 and CD117 was determined using fluorescence minus one (FMO) as a negative control (Fig. 4B).

Supplementary Fig. 1 shows five samples with typical NEC immunophenotype in MDS, MA, NC, and AC. The unified standard of CD105^+^NECs in each sample was 2500.

### Statistical analysis

RMFI was defined as the ratio between MFI of CD105^+^NEC and MFI of lymphocytes. Statistical analysis was performed using STATA software (version 14.0; Stata Corp LP, College Station, TX, USA). Visualization of the differential expression of a single variable between groups was performed using the "ggpubr" package in R software (version 4.1.0). Variables were considered significantly different between groups when *p* < 0.05. Receiver operating characteristic curves and AUCs were used to assess the diagnostic performance of variables for differential diagnosis. AUC > 0.800 was considered to have good diagnostic performance. The Youden index was used to calculate the best cut-off value to determine the corresponding sensitivity, specificity, accuracy, PPV, and NPV.

## Supplementary Information

Supplementary Figure 1.

## Data Availability

The original data presented in the study are included in the article; further inquiries can be directed to the corresponding author.
