# Supplementary material for: CD36 relative mean fluorescence intensity of CD105+ nucleated erythroid cells can be used to differentiate myelodysplastic syndrome from megaloblastic anemia
Source: Sci Rep. 2023 Jun 1;13:8930. doi: 10.1038/s41598-023-35994-9 (PMC10235022; doi:10.1038/s41598-023-35994-9)
Supplement: Supplementary file 1 — Supplementary Figure 1. [file 41598_2023_35994_MOESM1_ESM.pdf]

CD36 relative mean fluorescence intensity of CD105$^+$ nucleated erythroid cells can be used to differentiate myelodysplastic syndrome from megaloblastic anemia

Yan Lu[1], Xuya Chen[1] , Longyi Zhang[1]*

[1]Clinical Laboratory, Dongyang People's Hospital, 60 West Wuning Road, Dongyang 322100, Zhejiang, China

***Corresponding author:** Longyi Zhang, Clinical Laboratory, Dongyang People's Hospital, 60 West Wuning Road, Dongyang 322100, Zhejiang, China

E-mail: happy_zhang1y@163.com (LZ)

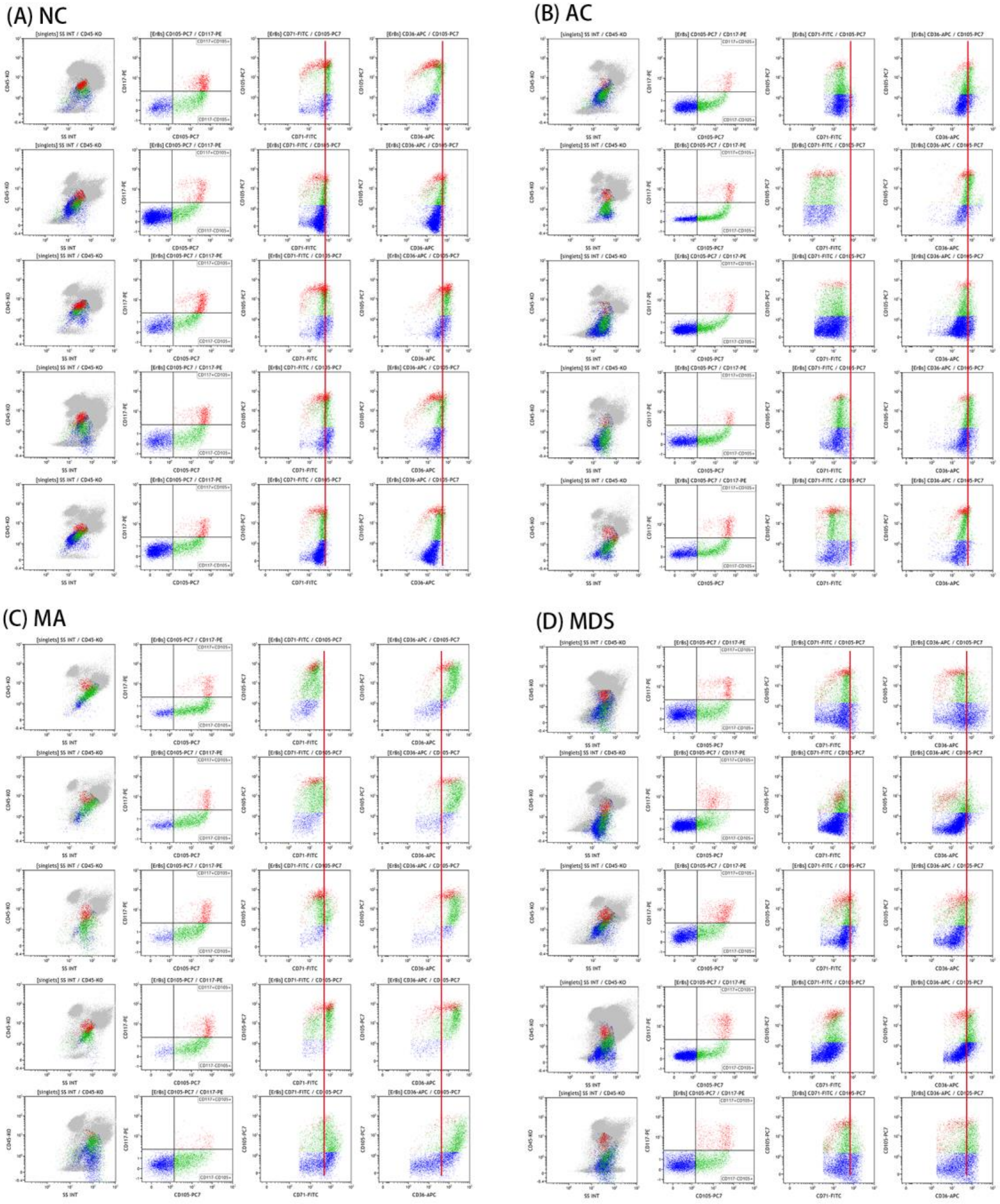

Supplementary Figure 1. Typical erythroid differentiation trajectory maps for MDS,MA,NC,AC. (A) NC; (B) AC; (C)MA; (D)MDS. Red cluster: CD117^{+}CD105^{+}nucleated erythroid cells; green cluster: CD117^{-}CD105^{+} nucleated erythroid cells; blue cluster: CD117^{-}CD105^{-} nucleated erythroid cells. MDS: myelodysplastic syndrome; MA: megaloblastic anemia; NC: normal controls; AC: anemic controls.
